# scalepopgen: Bioinformatic Workflow Resources Implemented in Nextflow for Comprehensive Population Genomic Analyses

**DOI:** 10.1093/molbev/msae057

**Published:** 2024-03-20

**Authors:** Maulik Upadhyay, Neža Pogorevc, Ivica Medugorac

**Affiliations:** Population Genomics Group, Department of Veterinary Sciences, LMU Munich, Martinsried 82152, Germany; Population Genomics Group, Department of Veterinary Sciences, LMU Munich, Martinsried 82152, Germany; Population Genomics Group, Department of Veterinary Sciences, LMU Munich, Martinsried 82152, Germany

**Keywords:** Nextflow, population genomics, signature of selection, workflows

## Abstract

Population genomic analyses such as inference of population structure and identifying signatures of selection usually involve the application of a plethora of tools. The installation of tools and their dependencies, data transformation, or series of data preprocessing in a particular order sometimes makes the analyses challenging. While the usage of container-based technologies has significantly resolved the problems associated with the installation of tools and their dependencies, population genomic analyses requiring multistep pipelines or complex data transformation can greatly be facilitated by the application of workflow management systems such as Nextflow and Snakemake. Here, we present scalepopgen, a collection of fully automated workflows that can carry out widely used population genomic analyses on the biallelic single nucleotide polymorphism data stored in either variant calling format files or the plink-generated binary files. scalepopgen is developed in Nextflow and can be run locally or on high-performance computing systems using either Conda, Singularity, or Docker. The automated workflow includes procedures such as (i) filtering of individuals and genotypes; (ii) principal component analysis, admixture with identifying optimal *K*-values; (iii) running TreeMix analysis with or without bootstrapping and migration edges, followed by identification of an optimal number of migration edges; (iv) implementing single-population and pair-wise population comparison-based procedures to identify genomic signatures of selection. The pipeline uses various open-source tools; additionally, several Python and R scripts are also provided to collect and visualize the results. The tool is freely available at https://github.com/Popgen48/scalepopgen.

## Introduction

Advancements in affordable sequencing technology coupled with relatively easy accessibility to high-performance computing (HPC) resources made it possible to generate and analyze hundreds of whole-genome sequences (WGS) in a relatively short time (Park and Kim 2016). As a result, recent studies have provided novel insights into the molecular basis of evolution and adaptation by applying population genomic techniques on a large number of WGS from diverse species. This includes uncovering widespread instances of adaptive introgression (Valencia-Montoya et al. 2020; Feng-hua et al. 2021), identification of candidate genes associated with local adaptation (Tigano et al. 2020; Ge et al. 2023), and disentangling the complex demographic history (Choudhury et al. 2020). Further, international efforts like the Vertebrate Genomes Project (Rhie et al. 2021) and the Earth BioGenome Project (Lewin et al. 2022), which aim to sequence and assemble high-quality reference genomes of eukaryotic species, will only accelerate the research in the field of population genomics.

The field of population genomics involves the development and application of complex statistical and computational methodologies (Charlesworth and Charlesworth 2017). Often these methodologies are either already implemented in the form of open-source software and/or occasionally, the researchers need to write their tools from scratch. A large number of open-source software require input data in a nonstandard format (other than variant calling format [VCF]); therefore, it is necessary to transform the data to use such software. Consequently, many researchers either lack the necessary resources or time to implement such analysis independently. These challenges are further amplified in a scenario where the output of one or multiple tools should be fed in as the inputs to another tool, forming a multistep workflow.

The following two examples demonstrate the abovementioned points. Application of ancestry estimation algorithm, *ADMIXTURE* tool (Alexander et al. 2009; Alexander and Lange 2011), requires that the researchers follow these steps: (i) filtering based on linkage disequilibrium (LD) (recommended), (ii) conversion of VCF to binary PED bed (BED) file format using *plink* (Purcell et al. 2007), (iii) run *ADMIXTURE* tool on the bed file, (iv) plot cross-validation (CV) error to identify a suitable value of *K*, (v) plot *Q*-matrices of *ADMIXTURE* outputs using tools such as *pong* (Behr et al. 2016) and *pophelper* (Francis 2017). Algorithms based on extended haplotype homozygosity (EHH) (Sabeti et al. 2002) calculations such as integrated haplotype score (iHS) (Voight et al. 2006) and cross-population EHH (XP-EHH) (Sabeti et al. 2007) require multiple steps as well: first, phasing the VCF with software packages such as *Beagle* (Browning and Browning 2007) and *Shapeit* (Delaneau et al. 2012); then appropriately transforming or formatting the VCF files to meet the requirements of the tools and packages like *selscan* (Szpiech and Hernandez 2014), *rehh* (Gautier et al. 2017), or *Hapbin* (Maclean et al. 2015). These examples also highlight the dependencies of the analyses on multiple software, which sometimes can be daunting to install, especially on HPC clusters due to the lack of administrative privileges. Further, updates and changes in the software and its dependencies may also limit the reproducibility of the research. To this end, containerization of such software and their dependencies using technologies like Conda, Singularity, and Docker have solved issues to some extent. Here, containerization refers to the process of bundling the tools and all their dependencies together so that they can run on any computational infrastructure. However, workflows for population genomic analyses that offer a comprehensive framework, integrating intermediary steps, and complex transformation are lacking. A few efforts have been made to address some of the abovementioned challenges. For instance, Webb et al. (2021) developed the *Pop-Gen pipeline platform*; it is a collection of modular functions written in Python programming language. These modular functions could either be run as stand-alone or combined to carry out various population genomic analyses including calculation of summary statistics, phasing of genotypes, and estimation of evolutionary history. However, some bioinformatic software and Python libraries that are required for these functions must be installed manually. Moreover, incorporating these functions with the batch scheduler on HPC clusters optimally requires some level of IT proficiency. Further, it also does not offer caching of task execution that allows the successfully completed task to be omitted and deployed for downstream analyses of the remaining tasks in case the workflow gets interrupted or canceled.

In recent years, the benefits of workflow management systems (Wratten et al. 2021) in terms of scalability, reproducibility, and deployability have been realized. Nextflow (Di Tommaso et al. 2017) is one such programming language that allows seamless integration and coordination among inputs and outputs of multiple tools, enabling workload scalability. Other advantages of Nextflow include portability, cross-perform functionality, and support for widely used job schedulers and orchestrators. All these features make the workflow portable from local computer to high-performance computer clusters, cloud services, and site workstations. It also ensures reproducibility by using the tools that are bundled with the container-based technology such as Docker, Singularity, and Conda (or Mamba).

Here, we employed the mentioned technologies to create *scalepopgen*, an easily scalable, portable, and reproducible tool that implements workflows for widely used population genomics analyses on genome-wide biallelic single nucleotide polymorphism (SNP) data. Specifically, these workflows have the features to filter the data, carry out the analyses to explore the genetic structure and phylogenetic relationships between the populations, and carry out the analyses to identify the genomic signatures of selection. These workflows can be run serially or parallel according to the user-defined criteria. After each analysis, the results are collected and the interactive visualization of these results is also generated; this feature can help the user with quick interpretation of the data. All the dependencies necessary to run the workflows are installed during the run-time of the analysis itself (a feature of Nextflow). Further, the tool can be extended by integrating the modules to run the analysis not currently included in the workflow.

## Materials and Methods

Note that, to maintain consistency, throughout the manuscript, the tools directly applicable in population genomic analyses are italicized.

### Design of the Pipeline

**Fig. 1.**
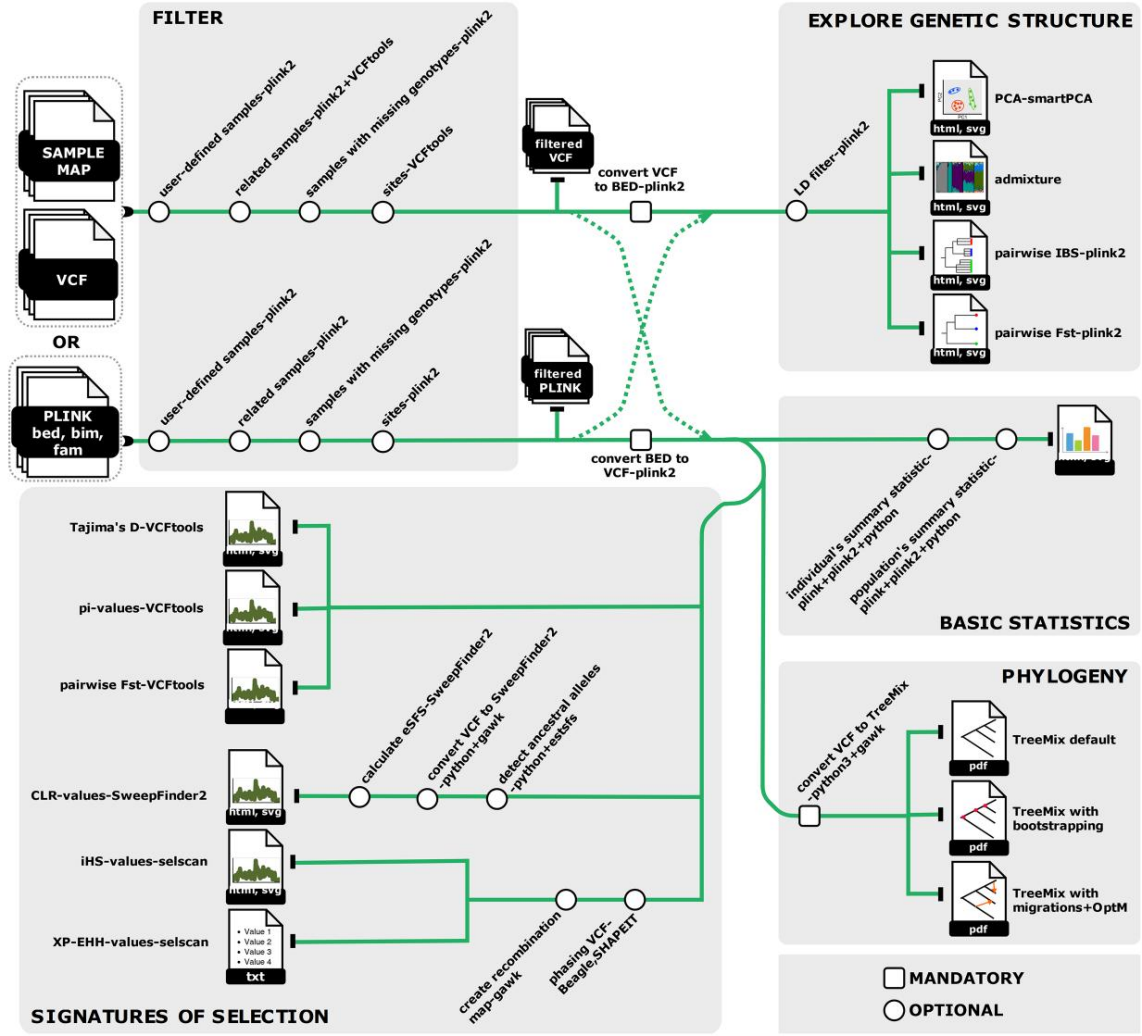
Overview of the workflows implemented in *scalepopgen*. The pipeline is implemented in the Nextflow workflow management system (Di Tommaso et al. 2017). It takes the CSV file containing the path to either VCF files or BED files as input and depending on the parameters set in a YAML configuration file runs the analysis and generates the results and its visualization. Note that the design of the workflow reflects the implementation in the current version (*scalepopgen* v 1.0.0).

*scalepopgen* is mainly developed using the Nextflow workflow management system (Di Tommaso et al. 2017), largely following the style of the nf-core domain-specific language 2 template. In this template, each process or step of the analysis is implemented as a module and each of these modules is further containerized using the software container technologies like Docker, Singularity, or Conda. The collection of such interconnected modules that are executed in a particular order forms the workflow. scalepopgen consists of several workflows that perform a diverse range of population genetic analyses (Fig. 1; supplementary tables S1 and S2, Supplementary Material online). For this purpose, it integrates frequently used open-source tools such as *Plink2 v2.00a3.7* (Purcell et al. 2007), *Vcftools v0.1.16* (Danecek et al. 2011), *Beagle v5.2* (Browning and Browning 2007), *Eigensoft v8.0.0* (Price et al. 2006), and many more (supplementary table S1, Supplementary Material online). Additionally, stand-alone tools were developed in R and Python whenever the appropriate open-source tools for the file transformation and/or the results compilation and its visualization could not be found. The execution of these subworkflows is controlled by a main script and a configuration file containing the parameters of the analyses.

The core parameters to run the workflow can be set in a YAML configuration file. These parameters control the types of analyses to be run and the argument to be used for the respective analysis. At a minimum, the user needs to provide as an input a comma-separated value (CSV) file containing either the path to the VCF files and their respective index files or the path to the BED files. In the case of VCF input, the files must be split by the chromosome/contigs, and an additional sample map file containing information about the samples and their respective populations must be provided. To assist the user in creating the configuration file and, subsequently, in running the workflow, an interactive Python-based command-line interface (CLI) is also supplied with the workflow.

Before carrying out the major population genetic analyses, the user has the option to filter samples or SNPs based on multiple criteria (Fig. 1; supplementary table S2, Supplementary Material online). For this purpose, *Plink v1.9*, *Plink2 v2.00a3.7* (Purcell et al. 2007), and *Vcftools v0.1.16* (Danecek et al. 2011) are used. Note that the filters are applied in two steps; site filtering is carried out after sample filtering. After the filtering steps, the workflow generates the reports of individual-based and population-based summary statistics and their interactive visualization. The report of individual sample-based statistics includes a total count of (i) heterozygous SNPs, (ii) SNPs with homozygous reference genotypes, (iii) SNPs with homozygous alternative alleles, (iv) transitions, and (v) transversions. The report of population-based summary statistics, composed according to the user-defined window, includes (i) the distribution of average minor allele frequency (MAF), and the distribution of average (ii) observed and (iii) expected heterozygosity. All the summary statistics are calculated using the same set of tools mentioned earlier in this paragraph and plotted using the custom Python scripts supplied with the workflow.

To explore the patterns of clustering among individuals and the genetic relationships between the populations, these approaches are implemented as workflows (supplementary fig. S1, Supplementary Material online): (i) principal component analysis (PCA), (ii) admixture analysis, (iii) identity by state (IBS)-distance-based clustering of samples, and (iv) *F*_ST_-distance-based clustering of populations. Note that the workflows are carried out as an end-to-end process. This means that all the steps—starting from converting or transforming the VCF to the custom input files as required by the respective tools, the analysis itself, and the interactive plotting of the results—are automatically carried out by the workflow. Several Python scripts are embedded within the workflows to (i) perform intermediate data conversion or transformation, (ii) collect the overall results, and (iii) plot the results interactively using the Python library Plotly v 5.18.0. Further, the user has the option to run only a subset of the analyses via settings in the configuration file. Additional features include user-defined pruning of the data based on LD parameters of *Plink2* (Chang et al. 2015), before supplying the input file to its respective analysis.

To investigate the genetic structure among the populations, PCA (supplementary fig. S1, Supplementary Material online) is implemented using *smartpca* of the *Eigensoft* (Patterson et al. 2006; Price et al. 2006) package (v 8.0.0). The Python scripts supplied with the workflow extract the percentage of genetic variance explained by each PC and plot the interactive visualization of the results. To estimate the individual ancestries, *ADMIXTURE* (v 7.0.2) (Alexander et al. 2009; Alexander and Lange 2011) tool is implemented in the workflow (supplementary fig. S1, Supplementary Material online). After performing the *ADMIXTURE* analysis, the Python scripts supplied with the workflow carry out the following processes in sequential order: (i) collect the log file of each run that is used to estimate ancestral contribution of user-defined common ancestors (also known as *K* values); (ii) extract the CV error written in those log files; (iii) based on the values of CV error, identify the most suitable value of *K* and plot its associated *Q*-matrix values in an interactive plot. Additionally, the input files necessary for interactive visualization of *ADMIXTURE* results using *PONG* (Behr et al. 2016) are also generated. Note that packages such as *SNPRelate* (Zheng et al. 2012) and *ADMIXTURE* (Alexander and Lange 2011) expect the chromosome ids in the form of integers. Therefore, if the parameter “--allow-extra-chr” is set to true, the workflow will recode the chromosome ids as the integers before supplying the bed files as input in such tools. The Python scripts for interactive visualization use the Bokeh v 3.2.1 Python library (https://docs.bokeh.org/en/latest/). The interactive plots have features such as hovering over the dots or the bars to get additional information about samples and controlling the dots or the bars to hide or to expose.

To investigate genetic relationships between populations, *Plink2* (Chang et al. 2015) is implemented to calculate the average pairwise *F*_ST_ (Weir and Cockerham 1984) distance between each pair of populations. Additionally, sample-based clustering is carried out from the square distance matrix generated with distances (as “1-ibs”). For this purpose, *Plink 1.9* (Chang et al. 2015) is implemented in the workflow. Next, the Python scripts included in the workflow carry out the following processes in sequential order: (i) calculate neighbor-joining (NJ) distances using the Python module *Biopython* (Cock et al. 2009) (v 1.80), (ii) plot interactive trees based on NJ distances using the Python modules *Toytree* (Eaton 2020) (v 2.0.5), and (iii) identify the list of the population whose samples form polyphyletic clusters; for this purpose, Python module *ete3* (v 3.1.3) (Huerta-Cepas et al. 2016), is used. The output from step (iii) can be useful to identify the outlier samples or samples that are mislabeled.

To visualize the results, the users can either supply the list of colors corresponding to each population or let the tool choose the colors representing each population randomly. Later, the allocated color–population pair is kept consistent across the visualization of the results of various analyses.

To explore the phylogenetic relationships between the populations, a separate workflow involving TreeMix analysis (supplementary fig. S2, Supplementary Material online) is implemented. An in-house Python script was developed to transform the VCF file to the input of the *TreeMix v 1.13* tool (Pickrell and Pritchard 2012). After performing the TreeMix analyses (with migration edges), the *OptM* (v 0.1.6) (Fitak 2021) package is used to summarize the results. Additionally, the consensus trees generated by the analyses are summarized using the *PHYLIP* (Felsenstein 1993) *CONSENSE* package (v 3.697). The resulting phylogenetic trees are plotted using the R Scripts provided with the TreeMix package or, in the case of the consensus trees, using the Python module *ete3* (v 3.1.3) (Huerta-Cepas et al. 2016).

To detect signatures of selection in the genome, the tool has several workflows (supplementary fig. S3, Supplementary Material online). In the case of the unphased data, the workflows are developed for the methods implemented in *vcftools* (v 0.1.16) (Danecek et al. 2011) and *SweepFinder2* (v 1.0.0) (DeGiorgio et al. 2016). Specifically, *Vcftools* is used to calculate Tajima's *D* (Tajima 1989) and *Pi* values for each population separately and additionally, Weir's *F*_ST_ (Weir and Cockerham 1984) between the pairs of populations or between one population at a time versus remaining samples of all other populations. Before running the *SweepFinder2* workflow, the user can opt to run the workflow that identifies the ancestral alleles using *est-sfs* (v 2.04) (Keightley and Jackson 2018), if the outgroup is present in the VCF files. Subsequently, this ancestral allele information is used to prepare *SweepFinder2* input files before computing the composite likelihood ratio (Kim and Stephan 2002) for each population. Before running the workflows for the methods implemented in *selscan* (v 2.0.2) (Szpiech and Hernandez 2014), the VCF files are phased using *BEAGLE* (v 5.2) (Browning and Browning 2007) or *SHAPEIT5* (v 5.1.1) (Hofmeister et al. 2023), and then iHS (Voight et al. 2006) and XP-EHH (Sabeti et al. 2002, 2007) are calculated for each population or each possible pair of populations, respectively. Finally, the Python scripts are used to produce interactive Manhattan plots for each analysis (except XP-EHH) and each population. Further, if the annotation resources for the species under investigation are present in the Ensembl database, then these plots also contain direct links to the location-based views of the regions that are identified as under selection based on the user-defined threshold.

### Case Study

Here, we demonstrate the application of *scalepopgen* to explore the genetic structure of a large number of cattle samples directly from the VCF files. To this end, we downloaded a data set of phased and imputed autosomal SNP genotypes extracted from 442 publicly available WGS representing 30 breeds (Gao et al. 2023). This data set lacked the detailed info and format fields of VCF. Using the CLI developed alongside the tool, we set parameters to (i) filter samples and sites based on criteria such as missingness of genotypes and MAF, (ii) obtain the summary statistics of samples as well as populations, (iii) run PCA and *ADMIXTURE* after filtering sites based on LD criteria, (iv) cluster the samples based on IBS distances, (v) cluster the population based on pairwise *F*_ST_ distances, and (vi) run TreeMix analysis considering bootstrapping as well as migration edges with iterations. The settings used in the YAML file are made available as supplementary note S1, Supplementary Material online. The following command was used to run the analyses:

nextflow run scalepopgen/-params-file test_full.yaml-profile singularity-resume

The input VCF files required to run this analysis are already available on the public repository (http://gong_lab.hzau.edu.cn/Animal_SNPAtlas/#!/download_cattle). The pipeline was run on the HPC server machine with 64 CPU cores and 2-TB RAM.

## Results

**Fig. 2.**
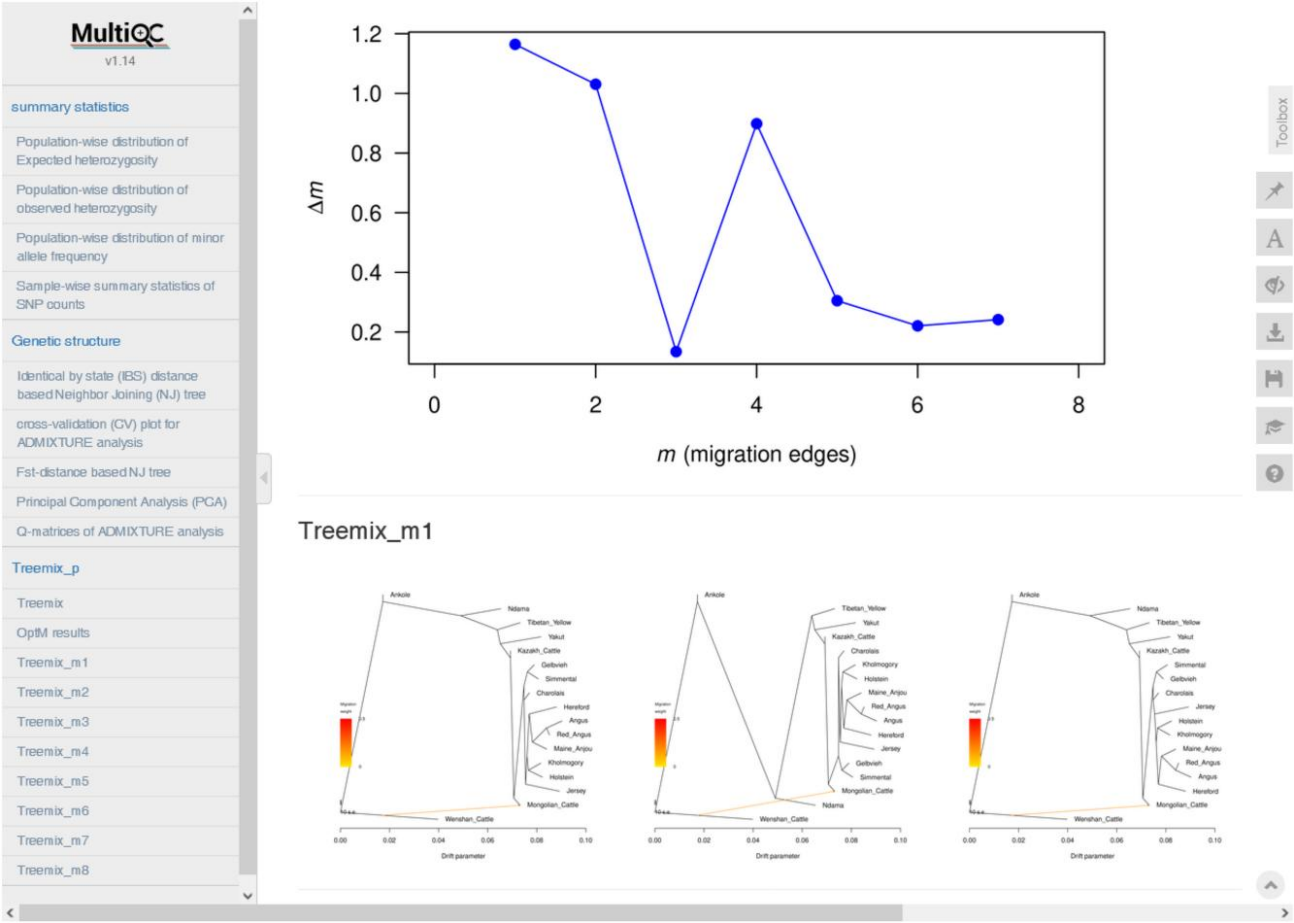
Preview of the visualization dashboard generated using *MultiQC*. It provides interactive visualization for (i) summary statistics generated using *Plink*, (ii) IBS-based clustering of the samples, (iii) *F*_ST_-distance-based clustering of the populations, (iv) PCA, (v) *ADMIXTURE*, and (vi) non-interactive visualization for *TreeMix* workflows. A complete interactive example can be assessed here: https://bioinf2305.github.io/scalepopgen_results/multiqc_report.html.

The entire pipeline was finished in ∼1.5 h, which was equivalent to ∼136 CPU hours. The HTML report containing the details about the computational resources and time used by each process can be accessed here: https://bioinf2305.github.io/scalepopgen_results/. At the end of the pipeline, *MultiQC* (v 1.19) (Ewels et al. 2016) is used to compile the visualization of the results of each analysis; this final HTML report (Fig. 2) can also be accessed on the aforementioned link.

**Fig. 3.**
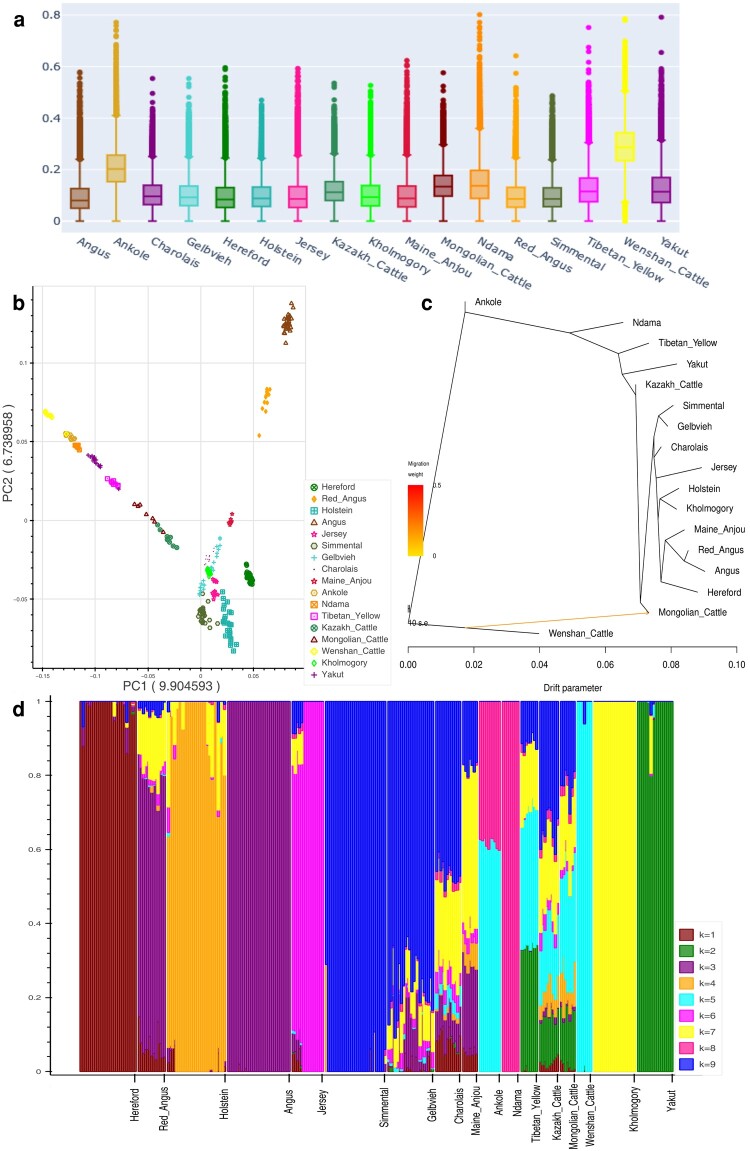
Overview of the plots generated by the workflows: a) distribution of average MAF calculated in 50-kb window in 17 cattle breeds, b) visualization of the PCA results generated by *Eigensoft* package, and c) ML-based phylogenetic tree with one migration edge as detected by the *TreeMix* analysis. d) Plotting of *Q*-matrix at *K* = 9. All used samples are available on NCBI SRA and their SNP genotypes were downloaded from the Animal-SNPAtlas (Gao et al. 2023).

The original phased data set consisted of ∼25 million SNPs genotyped in 442 samples (30 breeds). This data set was further reduced to 269 samples (17 breeds) and ∼23.4 million SNPs after applying sample- and site-filtering parameters. Population-based summary statistics such as MAF and observed and expected heterozygosity showed the highest values in Ankole and Wenshan cattle (Fig. 3a). Ankole is a sanga cattle breed that is a stable cross between zebu and taurine cattle, while Wenshan cattle are predominantly zebu with significant genetic contributions from taurine cattle.

To explore the genetic structure of the cattle population, the data set was further pruned to 129,357 SNPs using LD-based filtering. The PCA plot shows (Fig. 3b) the expected patterns with the samples belonging to the same breed clustering together. The first two PCs, explaining ∼10% and ∼7% of the total variations, respectively, separated the zebu-influenced breeds from the taurine breeds. Note that the Python script supplied with the workflow reads the eigenvalues of each PC from the output file and writes them as the *X* axis labels on the plot. The *ADMIXTURE* analysis suggested 9 as the most likely number of genetically distinct groups within the studied samples. It revealed the patterns of ancestry (Fig. 3d) for different cattle breeds similar to the previous studies (Kim et al. 2017; Tian et al. 2023). The patterns of population divergence as identified by the maximum-likelihood (ML) phylogenetic tree (Figs. 2 and 3c) using the *TreeMix* showed concordance with the results of PCA and *F*_ST_-distance-based population clustering. Wenshan cattle were used as an outgroup in the analysis. Ankole cattle occupied an intermediate position between zebu and taurine clades. In the taurine clade, first, Ndama diverged from the cattle breeds of Turano–Mongolian and European taurine. Subsequently, Turano–Mongolian cattle such as Mongolian cattle, Yakutian cattle, Tibetan yellow, and Kazakh cattle diverged from the core populations representing European taurine cattle. Interestingly, Kholmogory—allegedly a Turano–Mongolian breed—formed a sister clade with­ the Holstein cattle, which is in concordance with the previous studies (Yurchenko et al. 2018; Buggiotti et al. 2021). The optimal number of migration edges was detected as one by the second-order rate of change in likelihood (Δm) approach (Fig. 2) implemented in the *OptM* package. This migration edge was detected from Wenshan cattle to Mongolian cattle. This result is also in concordance with the previous study (Decker et al. 2014) showing migration edge from the cattle breeds of zebu lineage to Mongolian cattle in the TreeMix analysis.

## Discussion

The central objective behind the development of *scalepopgen* was to create workflows that provide easy accessibility to major population genomic analyses. These workflows are developed in Nextflow, and all the required packages are containerized; this arrangement provides advantages in terms of scalability and reproducibility of the analyses. Furthermore, almost all the analyses generate interactive visualization of the results. This is an extremely advantageous feature when dealing with thousands of samples because it is relatively easy to get additional information related to the results by either hovering over the points or clicking on the points on the plots. One of the features that can prevent the usability of this extensive workflow is the large number of parameters/arguments; to alleviate this issue, we also provide the CLI to create a YAML file required to run the workflow.

It is easy to extend this workflow by incorporating the additional tools. In fact, for future development, we aim to include tools that can carry out introgression analyses and demographic simulations. To this end, we invite the research community actively working in the field of population genomics to contribute additional modules or tools. The steps to contributing to the project are described on the GitHub page (https://github.com/Popgen48/scalepopgen). We welcome the suggestions concerning the improvements and extensions to this workflow.

## Supplementary Material

msae057_Supplementary_Data

## Data Availability

scalepopgen is hosted on GitHub under the popgen48 organization (https://github.com/Popgen48/scalepopgen) and released under MIT license. The results of the case study described in the paper are hosted at: https://bioinf2305.github.io/scalepopgen_results/.
