# Supplementary material for: scalepopgen: Bioinformatic Workflow Resources Implemented in Nextflow for Comprehensive Population Genomic Analyses
Source: Mol Biol Evol. 2024 Mar 20;41(4):msae057. doi: 10.1093/molbev/msae057 (PMC10994858; doi:10.1093/molbev/msae057)
Supplement: msae057_Supplementary_Data [file msae057_supplementary_data.pdf]

Supplementary Tables

Table S1. Overview of software and packages used in the scalepopgen pipeline. Note that Python or R packages listed not exhaustive and may change in the future versions.

**Tools (including Python or R packages)**	**Version**	**Purpose**
tabix	1.11	Indexing VCF files
gawk	5.1.0	Custom scripts/codes for the intermediate steps
VCFtools	0.1.16	Concatenating VCF files; calculating mean depth per sample; site filtering; sample filtering; removing custom samples; calculating pairwise Fst, Tajima's D, and nucleotide diversity
Plink	1.90b6.12	Updating chromosome IDs; calculating 1-IBS distances, counting population-based summary statistics such as minor allele frequency (MAF), observed heterozygosity, and expected heterozygosity
	2.00a3.7	Converting plink binary to VCF format; merging binary files; calculating global pairwise Fst; sample filtering; site filtering; LD-based filtering; removing custom samples; counting sample-based summary statistics
eigensoft	8.0.0	Running SmartPCA
Admixture	1.3.0	Running Admixture
Python	3.10	Development of custom scripts for the intermediate file transformation, collection of various results, and plotting
Biopython	1.81	Calculating Neighbor-Joining (NJ) distances
ete3	3.1.3	Identifying polyphyletic clusters based on 1-IBS distances
toytree	2.0.1	Interactive visualization of NJ trees
TreeMix	1.13	Constructing phylogenetic trees
R	4.3.2	Development of custom scripts for the file tranformation, collection of various results and its plotting
OptM	0.1.6	Determining optimal number of migrations in TreeMix analysis
est-sfs	2.04	Determining ancestral alleles
Beagle	5.2_21Apr21.304	Phasing genotypes
SweepFinder2	1.0.0	Calculating CLR
selscan	2.0.2	Calculating iHs and XP-EHH
MultiQC	1.19	Compilation of various results generated by the workflow
Imagemagick	7.1.0_44	Convert pdf to png and other image file formats.
bokeh	3.2.1	Interactive visualization of the results
plotly	5.18.0	Interactive visualization of the results

Table S2. Overview of the workflows implemented in the scalepopgen tool. Note that the name of the parameters and how it is invoked reflects the implementation in the current version (scalepopgen v 1.0.0).

**Workflow**	**How it is invoked**	**Overview of the capabilities**	**Tools used**
Sample filtering	--apply_indi_filters true	Filter samples based on relatedness, missingness of its genotypes, and custom individuals	Vcftools, plink, plink2
SNP filtering	--apply_snp_filters true	Filter SNPs based on all the criteria available in vcftools and Plink	Vcftools, plink2
Principal component analysis	--genetic_structure true --smartpca true	Transform vcf and plink-bed files to eigenstrat format, perform PCA, and plot the results	Plink2, eigensoft, and custom Python scripts are supplied with the tool.
ADMIXTURE	--genetic_structure true --admixture true	Transform vcf to plink-bed file format, perform admixture analysis, and plot the results	Plink2, ADMIXTURE, and custom Python scripts supplied with the tool
Fst distance-based NJ tree	--genetic_structure true --pairwise_global_fst true	Calculate the average pairwise distance between each pair of populations followed by the calculation of NJ distances and plot the tree	Plink2 and custom Python scripts supplied with the tool
Identity by state (IBS)-based NJ tree	--genetic_structure true --ibs_dist true	Calculate the average pairwise IBS distance between each pair of samples followed by the calculation of NJ distances group them and identify the population whose samples do not form the monophyletic clades	Plink2 and custom Python scripts are supplied with the tool.
Treemix	--treemix true	Transform vcf and plink-bed file format to treemix input	Treemix (including its Rscripts), OptM,

		format, run treemix analysis using the bootstrap approach, identify the optimal number of migration edges, and plot the results	and custom Python script supplied with the tool.
Identification of ancestral allele	--est_anc_alleles true	If the outgroup is present in the vcf file, the ancestry allele is identified	est-sfs and custom Python script supplied with the tool
Phasing	--beagle5 true or –shapeit5 true	Phase the genotypes	Beagle5, Shapeit5
Signature of selection using within-population measures	--pi_val true, --tajimas_d true, --fst_one_vs_all true –sweepfinder2 –ihs true	Calculate window-wise Tajima's D, pi values, Fst values. Calculate Composite likelihood ratio using sweepfinder2 and iHS values using selscan. Manhattan plot of the obtained results	Vcftools, Sweepfinder2, selscan and custom python scripts.
Signature of selection using pairwise-comparison	--pairwise_local_fst true --xpehh true	Calculate pairwise Fst value and pairwise XPEHH values for each marker or each window. Manhttan plot of the obtained results.	Vcftools, selscan and custom python scripts.

Table S3: List of 269 samples analyzed as a part of the case study presented in the paper; first column is the biosample ID of NCBI and the second column is its respective breed name.

SRS1338704	Ankole	SRS2165161	Mongolian_Cattle	SRS597618	Angus
SRS1496254	Ankole	SRS2165162	Mongolian_Cattle	SRS597619	Angus
SRS1496257	Ankole	SRS2505185	Wenshan_Cattle	SRS597620	Angus
SRS1496259	Ankole	SRS2505186	Wenshan_Cattle	SRS597621	Angus
SRS1496260	Ankole	SRS2505187	Wenshan_Cattle	SRS597622	Angus
SRS1496261	Ankole	SRS2505192	Wenshan_Cattle	SRS597623	Angus
SRS1496262	Ankole	SRS2505194	Wenshan_Cattle	SRS597624	Angus
SRS1496264	Ankole	SRS2505201	Wenshan_Cattle	SRS597625	Angus
SRS1496265	Ankole	SRS2505202	Wenshan_Cattle	SRS597626	Angus
SRS1496266	Ankole	SRS428704	Hereford	SRS597729	Holstein
SRS1512380	Ndama	SRS428705	Hereford	SRS597730	Holstein
SRS1512399	Ndama	SRS428708	Red_Angus	SRS597732	Holstein
SRS1512404	Ndama	SRS428709	Red_Angus	SRS597733	Holstein
SRS1512405	Ndama	SRS428710	Hereford	SRS597735	Holstein
SRS1512406	Ndama	SRS565374	Holstein	SRS597736	Holstein
SRS1512407	Ndama	SRS565646	Holstein	SRS597737	Holstein
SRS1512417	Ndama	SRS565647	Holstein	SRS597739	Holstein
SRS1512418	Ndama	SRS565648	Holstein	SRS597740	Holstein
SRS2165138	Tibetan_Yellow	SRS582840	Holstein	SRS597741	Holstein
SRS2165139	Tibetan_Yellow	SRS582855	Holstein	SRS597742	Holstein
SRS2165140	Tibetan_Yellow	SRS597595	Angus	SRS597743	Holstein
SRS2165142	Tibetan_Yellow	SRS597597	Angus	SRS597744	Holstein

SRS2165143	Tibetan_Yellow	SRS597598	Angus	SRS597746	Holstein
SRS2165144	Tibetan_Yellow	SRS597601	Angus	SRS597747	Holstein
SRS2165145	Tibetan_Yellow	SRS597602	Angus	SRS597748	Holstein
SRS2165146	Tibetan_Yellow	SRS597603	Angus	SRS597749	Holstein
SRS2165147	Kazakh_Cattle	SRS597604	Angus	SRS597750	Holstein
SRS2165148	Kazakh_Cattle	SRS597605	Angus	SRS597751	Holstein
SRS2165149	Kazakh_Cattle	SRS597606	Angus	SRS597752	Holstein
SRS2165150	Kazakh_Cattle	SRS597607	Angus	SRS597753	Holstein
SRS2165151	Kazakh_Cattle	SRS597608	Angus	SRS597755	Holstein
SRS2165152	Kazakh_Cattle	SRS597609	Angus	SRS597756	Jersey
SRS2165153	Kazakh_Cattle	SRS597610	Angus	SRS597757	Jersey
SRS2165154	Kazakh_Cattle	SRS597611	Angus	SRS597758	Jersey
SRS2165155	Kazakh_Cattle	SRS597612	Angus	SRS597759	Jersey
SRS2165156	Mongolian_Cattle	SRS597613	Angus	SRS597760	Jersey
SRS2165157	Mongolian_Cattle	SRS597614	Angus	SRS597761	Jersey
SRS2165158	Mongolian_Cattle	SRS597615	Angus	SRS597762	Jersey
SRS2165159	Mongolian_Cattle	SRS597616	Angus	SRS597763	Jersey
SRS2165160	Mongolian_Cattle	SRS597617	Angus	SRS597764	Jersey
SRS597765	Jersey	SRS628366	Gelbvieh	SRS629691	Charolais
SRS597766	Jersey	SRS628367	Gelbvieh	SRS629692	Maine_Anjou
SRS597767	Jersey	SRS628368	Gelbvieh	SRS629693	Gelbvieh
SRS597768	Jersey	SRS628369	Charolais	SRS629694	Hereford
SRS597769	Jersey	SRS628370	Charolais	SRS631187	Red_Angus
SRS597770	Jersey	SRS628371	Charolais	SRS631188	Red_Angus

SRS597771	Simmental	SRS628372	Charolais	SRS631190	Hereford
SRS597772	Simmental	SRS628373	Red_Angus	SRS631191	Hereford
SRS597773	Simmental	SRS628374	Red_Angus	SRS631192	Hereford
SRS597774	Simmental	SRS628376	Charolais	SRS631193	Maine_Anjou
SRS597775	Simmental	SRS629092	Hereford	SRS631194	Hereford
SRS597776	Simmental	SRS629094	Hereford	SRS631195	Hereford
SRS597777	Simmental	SRS629095	Hereford	SRS631196	Red_Angus
SRS597778	Simmental	SRS629097	Hereford	SRS631197	Gelbvieh
SRS597780	Simmental	SRS629099	Hereford	SRS631198	Hereford
SRS597781	Simmental	SRS629100	Gelbvieh	SRS631207	Charolais
SRS597782	Simmental	SRS629321	Charolais	SRS631208	Hereford
SRS597785	Simmental	SRS629325	Gelbvieh	SRS631210	Maine_Anjou
SRS597786	Simmental	SRS629326	Gelbvieh	SRS631211	Hereford
SRS597787	Simmental	SRS629334	Charolais	SRS631212	Maine_Anjou
SRS597788	Simmental	SRS629338	Red_Angus	SRS631213	Hereford
SRS597789	Simmental	SRS629339	Hereford	SRS631215	Maine_Anjou
SRS597791	Simmental	SRS629340	Hereford	SRS631216	Hereford
SRS597792	Simmental	SRS629341	Red_Angus	SRS631218	Hereford
SRS597794	Simmental	SRS629669	Gelbvieh	SRS631221	Gelbvieh
SRS597795	Simmental	SRS629671	Maine_Anjou	SRS631223	Hereford
SRS597796	Simmental	SRS629672	Red_Angus	SRS631224	Hereford
SRS597797	Simmental	SRS629673	Gelbvieh	SRS631225	Hereford
SRS597798	Simmental	SRS629674	Gelbvieh	SRS631226	Hereford
SRS597799	Simmental	SRS629675	Gelbvieh	SRS631230	Charolais

SRS597808	Simmental	SRS629676	Gelbvieh	SRS641736	Red_Angus
SRS597809	Simmental	SRS629678	Maine_Anjou	SRS641737	Red_Angus
SRS597811	Simmental	SRS629679	Gelbvieh	SRS641741	Red_Angus
SRS597812	Simmental	SRS629681	Gelbvieh	SRS641742	Gelbvieh
SRS597813	Simmental	SRS629682	Gelbvieh	SRS6910385	Kholmogory
SRS614728	Angus	SRS629683	Gelbvieh	SRS6910386	Kholmogory
SRS628362	Hereford	SRS629684	Gelbvieh	SRS6910387	Kholmogory
SRS628363	Gelbvieh	SRS629686	Charolais	SRS6910388	Kholmogory
SRS628364	Gelbvieh	SRS629687	Charolais	SRS6910389	Kholmogory
SRS6910390	Kholmogory	SRS6910407	Yakut	SRS6910405	Yakut
SRS6910391	Kholmogory	SRS6910408	Kholmogory	SRS6910406	Yakut
SRS6910392	Kholmogory	SRS6910409	Yakut	SRS6910401	Yakut
SRS6910393	Kholmogory	SRS6910411	Yakut	SRS6910402	Yakut
SRS6910394	Kholmogory	SRS6910412	Yakut	SRS6910403	Yakut
SRS6910395	Kholmogory	SRS6910414	Yakut	SRS6910404	Yakut
SRS6910396	Kholmogory	SRS6910415	Yakut	SRS6910421	Kholmogory
SRS6910397	Kholmogory	SRS6910417	Yakut	SRS6910422	Kholmogory
SRS6910398	Yakut	SRS6910418	Yakut	SRS6910423	Kholmogory
SRS6910399	Yakut	SRS6910419	Kholmogory	SRS6910424	Kholmogory
SRS6910400	Yakut	SRS6910420	Kholmogory		

Supplementary Figures

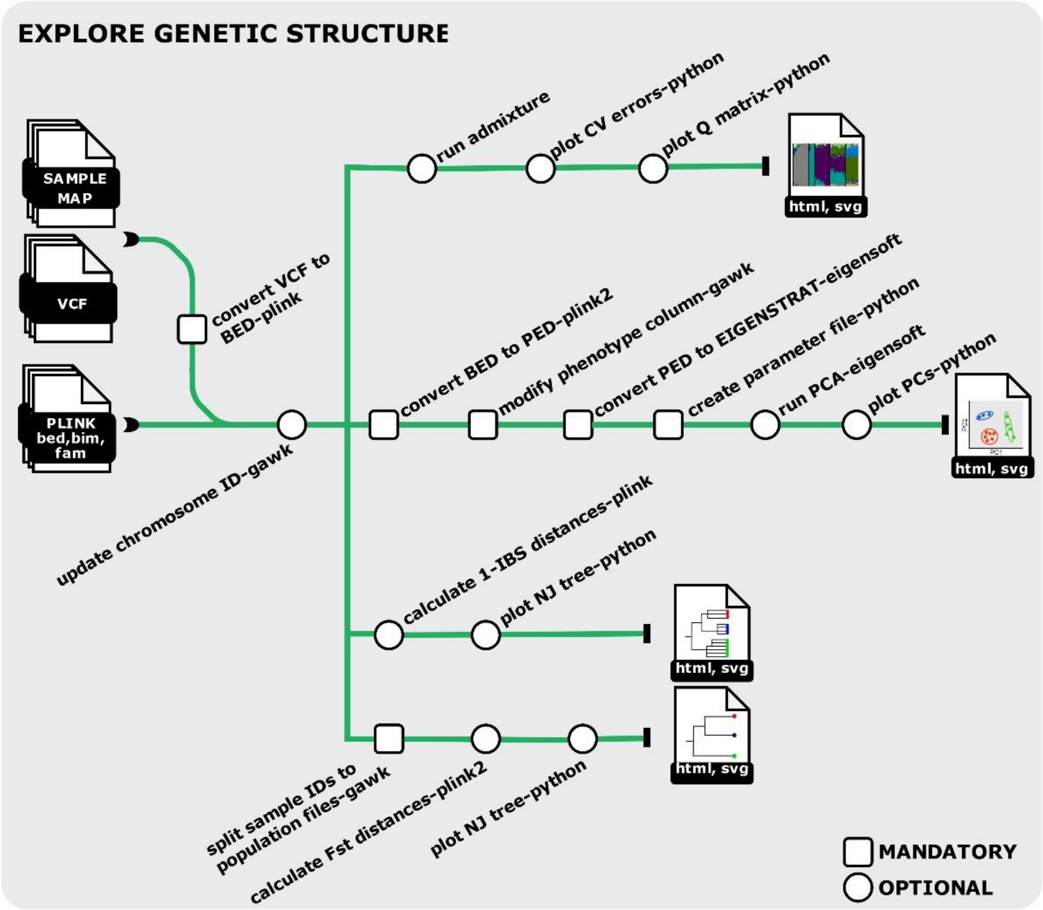

Fig. S1. Overview of the workflows that implement the analyses to explore the genetic structure of the population. Broadly, the tool has workflows to carry out these analyses: (i) PCA, (2). ADMIXTURE, (3) calculate pairwise 1-IBS distance among the samples and cluster them using NJ approach, (4) calculate pairwise Fst distance among the population and cluster them using NJ approach. Note that the design of the workflow reflects the implementation in the current version (scalepopgen v 1.0.0).

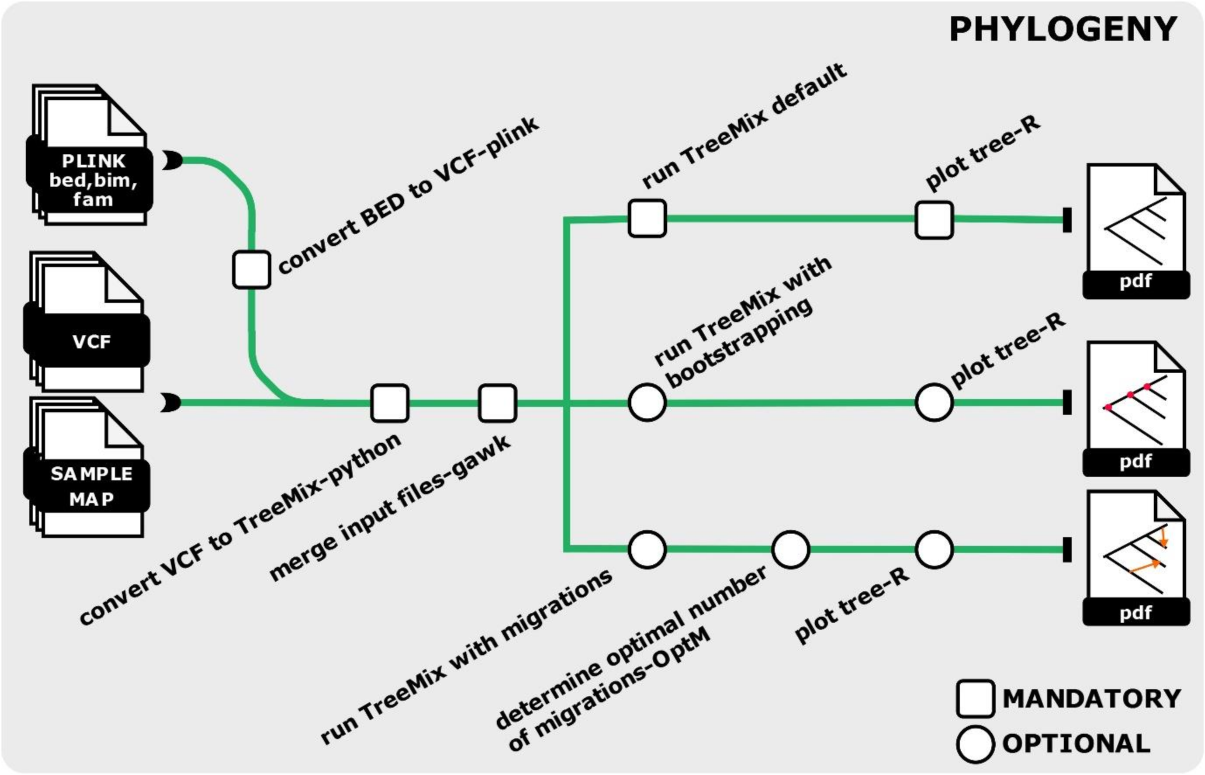

Fig. S2. Overview of the workflow that implements treemix analyses. This workflow includes options to generate the consensus tree using the boostrap approach as well as identify the optimal migration edges after running the treemix with migration edges. Note that the design of the workflow reflects the implementation in the current version (scalepopgen v 1.0.0).

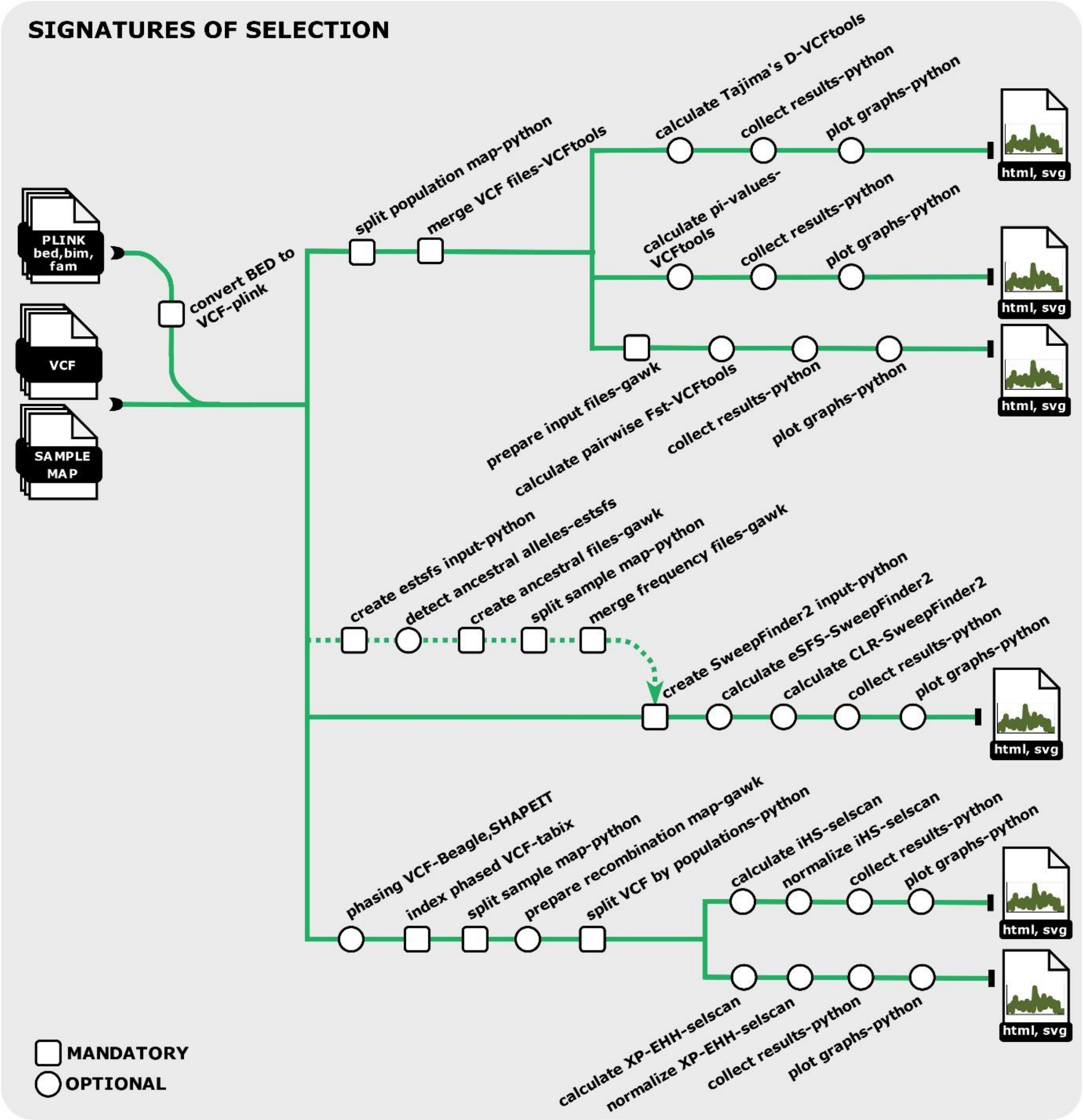

Fig. S3. Overview of the workflows to identify genomic signatures of selection. Broadly, the pipeline has workflows to run signature of selection analyses implemented in vcftools, sweepfinder2 and selscan. Note that the design of the workflow reflects the implementation in the current version (scalepopgen v 1.0.0).

Supplementary note S1

Following are the parameters set in YAML file to run the analyses described in the paper:

input = 'https://raw.githubusercontent.com/Popgen48/scalepopgen-test/main/workflow_test_files/test_full/test_input.csv'

rem_indi = 'https://raw.githubusercontent.com/Popgen48/scalepopgen-test/main/workflow_test_files/test_full/samples_to_be_removed.txt'

sample_map= 'https://raw.githubusercontent.com/Popgen48/scalepopgen-test/main/workflow_test_files/test_full/sample_map.map'

apply_indi_filters = true

apply_snp_filters = true

admixture = true

end_k = 20

genetic_structure = true

ibs_dist = true

ld_filt = true

ld_step_size = 100

ld_window_size = 500

indiv_summary = true

outdir = "test_paper_out"

pairwise_global_fst = true

r2_threshold = 0.05

smartpca = true

k_snps = 500

maf = 0.01

outgroup = "Wenshan_Cattle"

rand_k_snps = true

treemix = true

n_mig = 8

n_iter = 3

king_cutoff = 0.0884

mind = 0.05

max_missing = 0.05
